# The Effectiveness of Mindfulness-Based Stress Reduction on Psychological Distress and Cognitive Functioning in Patients with Multiple Sclerosis: a Pilot Study

**DOI:** 10.1007/s12671-017-0701-6

**Published:** 2017-03-28

**Authors:** Roos J. Blankespoor, Melanie P.J. Schellekens, Sandra H. Vos, Anne E.M. Speckens, Brigit A. de Jong

**Affiliations:** 1 Department of Medical Psychology, Radboud University Medical Center, PO Box 9101, 6500 HB, Nijmegen, the Netherlands; 2 Department of Psychiatry, Radboud University Medical Center, PO Box 9101, 6500 HB, Nijmegen, the Netherlands; 3 Department of Neurology, VUmc MS Center Amsterdam, VU University Medical Center, PO Box 7057, 1007 MB, Amsterdam, the Netherlands; 4 Department of Neurology, Radboud University Medical Center, PO Box 9101, 6500 HB, Nijmegen, the Netherlands

**Keywords:** Mindfulness-based stress reduction, Multiple sclerosis, Cognitive functioning, Psychological distress, Quality of life

## Abstract

Patients with multiple sclerosis (MS) often suffer from psychological distress and cognitive dysfunctioning. These factors negatively impact the health-related quality of life. Only recently behavioral therapeutic approaches are being used to treat psychological distress in MS. The aim of the present pilot study was not only to investigate the effectiveness of mindfulness-based stress reduction (MBSR) on psychological distress but also to explore whether it can improve cognitive functioning among patients with MS. Outpatients of the MS Center of the Radboud University Medical Center (Radboudumc) were invited to participate in an MBSR training. Psychological and cognitive measures were administered pre- and post-intervention. Twenty-five MS patients completed the MBSR training and psychological measures, of which 16 patients completed the cognitive tests. Significant improvements were found in depressive symptoms, quality of life, fatigue, mindfulness skills, and self-compassion. Of the cognitive tests, performance on a visual spatial processing test significantly improved after the intervention. Overall, this pilot study showed promising results of the effects of MBSR on reducing psychological distress, and it suggests MBSR might improve cognitive functioning in MS patients. Future randomized controlled trials should be conducted to confirm the possible effectiveness of MBSR—and its long-term effects—on psychological and cognitive functioning in MS patients.

## Introduction

Psychological distress and cognitive dysfunction are common clinical characteristics in multiple sclerosis (MS), and have significant impact on quality of life (Amato et al. 2001; Arnett et al. 2008; Chiaravalloti and DeLuca 2008; Feinstein 2006; Kern et al. 2009). During all stages of this disease, patients report psychological distress, partly due to disease-related factors, i.e., its unpredictable disease course and physical impairments (Amato et al. 2001; Feinstein 2006). Depression is a common concomitant among patients with MS, with a prevalence rate up to 50% (Arnett et al 2008; Feinstein, 2011; Kern et al. 2009). In addition, fatigue—both physical and cognitive—has been reported by 90% of patients (Amato et al. 2001; Chiaravalloti and DeLuca 2008).

Cognitive impairments in memory, information processing speed, executive functioning, (complex) attention, and verbal fluency are a common feature of MS, with a prevalence rate between 43 and 70% (Chiaravalloti and DeLuca, 2008; Feinstein et al. 2013; Winkelmann et al. 2007). Although symptoms vary substantially between patients with MS, they can be subtle and generally result in progression over time. Because of psychological distress, fatigue, depression, and cognitive deficits, in 40 to 60% of all patients the overall quality of everyday and professional life is severely affected (Chiaravalloti and DeLuca, 2008; Feinstein et al. 2013).

So far most MS research has focused on medication treatment of somatic symptoms. Only recently behavioral therapeutic approaches considered treatment of psychological distress in MS, mostly by using cognitive behavioral therapy (CBT) (Thomas et al. 2006; Winkelmann et al. 2007). CBT consists of individual sessions with a therapist, using rather complex verbally based techniques (Beck 1995). A more experiential approach, as used in mindfulness-based interventions (MBI), might be more suitable for MS patients who often suffer from cognitive impairments. Recent studies have indicated MBIs are a promising choice in treating psychological functioning in MS patients (Bogosian et al. 2015; Grossman et al. 2010).

One of the most often studied MBI is mindfulness-based stress reduction (MBSR). MBSR was developed by Jon Kabat-Zinn (1990), who defined mindfulness as intentionally paying attention to present moment experiences in a non-judgmental way. Studies have indicated that MBIs, and MBSR in particular, affect psychological functioning and may improve anxiety, depression, stress, and quality of life in clinical and healthy populations (Gotink et al. 2015; Goyal et al. 2014; Khoury et al. 2013). In addition, two recent randomized controlled trials reported also improvement in MS patients on measures of pain, anxiety (Bogosian et al. 2015), depression, fatigue, and quality of life after MBI participation (Bogosian et al. 2015; Grossman et al. 2010). Bogosian et al. (2015) assessed the effectiveness of a special Skype distant-delivered MBI, which was based on mindfulness-based cognitive therapy (MBCT), which integrates aspects of CBT and MBSR. Grossman et al. (2010) investigated the effects of an MBI closely following the structure of MBSR.

To our knowledge, no studies have systematically investigated the effects of MBIs on cognitive functioning as measured with a valid neuropsychological test protocol in patients with MS. Chiesa et al. (2011) systematically reviewed the effects of MBIs on cognitive abilities, such as attention, memory, and executive functions. They concluded that MBIs have the potential to improve cognitive abilities in healthy and depressed participants. In the studies that included patients with chronic pain and traumatic brain injury, no improvements were found after MBI participation. Due to the presence of cognitive impairments and psychological distress in the majority of MS patients, which negatively impact daily life, we expect MS patients to benefit from MBSR on both psychological and cognitive functioning. The aim of the current study was not only to investigate whether MBSR improves psychological functioning and quality of life, but also to explore whether it can improve objective cognitive functioning among patients with MS.

## Method

### Participants

About 150 outpatients of the Radboud University Multiple Sclerosis Center, Nijmegen, the Netherlands attended a study day on mindfulness for MS patients. During this day, around a fifth of all attendees expressed interest in MBSR participation. Patients were included when they had a sufficient understanding of the Dutch language. As this was a pilot study, no selection criteria for MS disease subtype and disease duration were used. The local medical ethics committee indicated that no formal approval was required as the study was an uncontrolled study of an intervention already on offer in clinical care, and the administration of questionnaires was considered to be routine clinical outcome monitoring (nr. 2014/218).

### Procedure

Postal paper-pencil questionnaires were sent to participants approximately 1 week before and 1 week after the completed MBSR intervention. The cognitive tests were administered at the Radboud University Multiple Sclerosis Center by two clinically trained neuropsychologists interns. If possible, patients were tested pre- and post-intervention by the same neuropsychologist. Cognitive tests were conducted 1 week preceding the intervention and 2 weeks after the final session, so the time interval between pre- and post-test ranged from 8 to 10 weeks. All tests were assessed at the same fixed order under standard test circumstances. The total duration of each cognitive assessment ranged from 90 to 120 min, including at least one break and if required more. No monetary reward was given to the patients, as they received mindfulness training without any additional costs.

#### Intervention

MBSR is based on the mindfulness training program developed by Jon Kabat-Zinn (1990) and consists of eight weekly 2.5-h group sessions, a six-hour silence-day and daily take home exercises of 45 min. Sessions consist of meditation practices (such as the body scan, gentle yoga, sitting, and walking meditation), didactic teaching on stress and sharing experiences with one another. Two professionally trained mindfulness teachers who fulfilled the criteria for teaching mindfulness-based interventions trained the groups (Crane et al. 2012).

### Measures

#### Psychological Self-Report Questionnaires

The Beck Depression Inventory (BDI) is a 21-item questionnaire assessing the level of depressive symptoms, scores range from 0 to 63 (Beck et al. 1996). The BDI has been shown to have good psychometric properties, including good internal consistency (.73–.92). Multiple Sclerosis Quality of Life-54 (MSQL-54) measures quality of life with 36 generic questions from the Short Form 36-item Health Survey, and 18 additional items that are specific to MS (Vickrey et al. 1995). The 54 items can be divided into 12 multi-item scales. Two composite scores, physical, and mental health, were calculated as a weighted sum of selected scales. The MSQL-54 has been shown to have good internal consistency in a MS population (.75–.96). Checklist Individual Strength—Fatigue (CIS-F) is a self-report measure with 8 items scored on a 7-point Likert scale and assess fatigue severity over the last 2 weeks (Vercoulen et al. 1994). Scores range from 8 to 56. A score of ≥35 is indicated as severely fatigued The CIS has a good internal consistency (.90), discriminative validity and is sensitive to change (Vercoulen et al. 1994). Five Facet Mindfulness Questionnaire (FFMQ) is a 39-item questionnaire consisting of the following subscales: observing, describing, act with awareness, non-judging of inner experiences, and non-reactivity to inner experiences (Baer et al 2006). It appears to be psychometrically promising, each facet showing good internal consistency (.81–.86) and expected correlations with several other variables. Consistent with predictions, most mindfulness facets are significantly related to meditation experience and to psychological symptoms and well-being (Baer et al. 2006; Baer et al. 2008). Self-Compassion Scale (SCS) (Neff, 2003) measures self-compassion with 26 items divided over six subscales: self-kindness versus self-judgment, common humanity versus isolation, and mindfulness versus over-identification. Internal consistency is high (.92). Self-compassion is significantly correlated with positive mental health outcomes such as less depression and anxiety and greater life satisfaction. There is also evidence for the discriminant validity of the scale, with regard to self-esteem measures (Neff 2003).

#### Cognitive Measures

Six neuropsychological tests available in the Dutch language that cover the cognitive domains that are most commonly impaired in MS patients were selected and matched to the validated minimal assessment of cognitive functions in MS (MACFIMS) (Benedict et al. 2006) and the Dutch Guidelines for MS research (Nederlandse Vereniging voor Neurologie 2011). To prevent learning effects at post-test parallel test-versions were used if available. To assess subjective memory complaints, a corresponding questionnaire was added to the cognitive tests. The *Multifactorial Meta Memory Questionnaire (MMQ)* paper-pencil questionnaire measures subjective self-reported memory (Van der Werf and Vos 2011) within three dimensions: memory-contentment (18 items), daily forgetfulness (20 items), and memory strategy use (19). Items can be rated on a 5-point scale. Higher scores indicate more contentment (≥27), less daily forgetfulness (≥44), and more use of memory strategies (≥37). Examples of questions are, respectively: ‘I have confidence in my ability to remember things’; How often do you forget a daily appointment?’; ‘How often do you put something on a prominent place to remind you to do something?’ Internal consistency has been indicated as high in both clinical and non-clinical populations (Van der Werf and Vos 2011). The Dutch version Rey Auditory Verbal Learning Test (RAVLT) measures new learning and verbal memory (Van Der Elst et al. 2005). This test requires patients to remember and recall 15 verbally presented, unrelated words in five consecutive learning trials. After 20 min, there is a delayed free recall, which measures memory decline. The outcome measures are the average number of remembered and recalled words. The Location Learning Task (LLT) measures visuospatial memory (Kessels et al. 2006). Ten everyday objects printed on different locations on a 5 × 5 grid are shown for 15 s to the patients. Subsequently, the stimulus card is covered by a blank grid, and patients have to remember and to relocate the objects. There are five consecutive learning trials, and a delayed recall after 20 min. The displacement score is the total sum of errors for each (incorrect) replaced picture. Thus, a displacement score of “0” indicates a perfect score. The learning index displays the average measure for the relative difference in performance between trials, and thereby, measures overall improvement or aggravation. The LLT has been validated in clinical groups (Kessels et al. 2006). The Paced Auditory Serial Addition Test (PASAT) measures processing speed (Tombaugh 2006). This test requires patients to listen to a series of 61 auditory presented digits between 1 and 6 at a 3.2-s interval rate. Patients have to add and recall each following digit with the one immediately preceding it. So for example, if the digits 3, 6, and 2 are presented, the patient has to respond with “9” (3 + 6) and “8” (6 + 2). The test is preceded with a short training-trial of ten digits. The original test consists of five fixed inter-stimulus intervals (3.2, 2.4, 2.0, 1.6, and 1.2 s) that increase in difficulty. We choose for the 3.2 s interval because (1) from clinical experience, this task has been shown to be very difficult for MS patients since they exhibit a lower speed of information processing, and (2) applying all intervals would be too time-consuming. The total score is the sum of correct repeated digits. The PASAT shows high internal consistency (Tombaugh 2006). The digit span test (taken from Wechsler Adult Intelligence Scale-lll) measures working memory and attention (Lezak 2004). This test requires patients to immediately repeat verbally presented series of digits increasing in length (from 2 to 9 digits), first forwards and then backwards. The total score consists of the correct repeated series of digits. The Letter-number sequencing test (taken from Wechsler Adult Intelligence Scale-lll) measures executive function (Lezak 2004). Patients have to reorder randomly mixed numbers and letters by first naming the digits in increasing number, followed by the letters in alphabetical order (from 2 to 8 items). The total score consists of the correct repeated series of digits and letters. The letter fluency test measures language and semantic memory (Lezak 2004). This test requires patients to generate as many words with a given letter in 1 min, in three consecutive trials with three different letters (pretest D-A-T, post-test K-O-M). The total score is the sum of all produced words.

### Data Analyses

This study was an uncontrolled within-subject pilot study. Changes from pre- to post-intervention were calculated with paired-sample *t* tests considering a two-tailed significance level of .05. Performance on the cognitive tests was compared and interpreted with standardized non-clinical population measures. For the PASAT, *z* scores were calculated from the sample, as no standardized scores were available. Deviation for a single test for all patients individually was defined as a *z* score < −2.00 below standardized population measures. For all measures, the 95% confidence interval (95% CI) for the mean difference was reported. To indicate the magnitude of change from pre-to post-intervention, effect sizes (Cohen’s *d*) were calculated.

## Results

### Participants

Thirty-one patients signed up to voluntarily participate in the MBSR program and were offered questionnaires pre- and post-intervention. Twenty-five patients (19% dropout) completed at least four 2.5-h sessions, and all questionnaires pre- and post-intervention (mean age *M =* 52.64, SD = 10.67, 84% woman) (Table 1). Of the 31 patients who signed up to participate, only 23 patients were offered cognitive tests (groups 1 and 2). Sixteen patients (30% dropout) completed cognitive tests pre- and post-intervention (mean age *M =* 55.19, SD = 6.53, 75% woman). The high dropout rate (*n* = 7, 5 women) at cognitive post-intervention testing was mainly caused by practical problems (i.e., traveling distance), lack of time and motivation, and psychological problems. Two patients refused to participate after two and five mindfulness sessions.

**Table 1:** Demographic and clinical characteristics of the patients that completed questionnaires (*n* = 25), and the patients that received and completed additional cognitive tests (*n* = 16)

	Questionnaires (*n* = 25)	Cognitive measures (*n* = 16)
Age (years), *M* (SD)	52.64 (10.67)	55.19 (6.53)
Gender, woman, *n* (%)	21 (84)	12 (75)
Education level (*n* = 19), *M* (SD)	5.42 (.84)	5.31 (.87)
Type of MS, *n* (%)		
Relapsing-remitting MS	7 (28)	5 (31)
Secondary progressive MS	8 (32)	7 (44)
Primary progressive MS	4 (16)	4 (25)
Unknown	6 (24)	–
Years since diagnosis, *M* (SD)	14.81 (9.92)	
Relapsing-remitting MS	9.63 (3.96)	
Secondary progressive MS	20.67 (12.25)	
Primary progressive MS	12.00 (5.83)	

Note: education level is rated according to Verhage (1964) range 1–7: *1* less than 6 years of education, *7* university degree

### Psychological Functioning

With regard to the self-report questionnaires, significant improvements from pre- to post-intervention were found in depressive symptoms (*t*(24) = −3.25, *p* = .003, *d* = .63), the physical (*t*(24) = 6.16, *p* < .001, *d* = 1.20), and emotional (*t*(24) = 9.04, *p* < .001, *d* = .1.45) domains of quality of life and fatigue (*t*(24) = −2.87, *p* = .008, *d* = .48). Patients also reported significant improvements in mindfulness skills (*t*(23) = 3.82, *p* = .001, *d* = .88), specifically the observing (*t*(23) = 3.96, *p* = .001, *d* = .73), describing (*t*(23) = 2.50, *p* = .020, *d* = .38), and non-reactivity to inner experiences (*t*(23) = 4.10, *p* < .001, *d* = .97) facets. Self-compassion also improved from pre- to post-intervention (*t*(24) = 3.25, *p* = .014, *d* = .48), specifically an increase in the subscales self-kindness (*t*(24) = 2.64, *p* = .014, *d* = .50), and a decrease in over-identification (*t*(24) = −2.79, *p* = .010, *d* = .45) (Table 2).

**Table 2:** Effects of MBSR on psychological measures from pre- to post-intervention in 25 MS patients

	Pre-intervention		Post-intervention		*p*	Cohen’s *d*	95% CI
	*M*	SD	*M*	SD			
Depressive symptoms (BDI)	12.6	6.3	9.0	5.1	.003	0.63	−5.89, −1.31
Quality of life (MSQoL-54)							
Physically	49.5	14.4	67.4	15.5	<.001	1.20	11.92, 23.92
Mentally	51.0	15.7	72.9	14.4	<.001	1.45	16.89, 26.89
Fatigue (CIS-F)	41.2	8.6	36.6	10.3	.008	0.48	−7.77, −1.27
Mindfulness skills (FFMQ)	127.1	13.6	139.0	13.5	.001	0.88	−5.47, 18.37
Observing	27.3	5.5	30.6	3.6	.001	0.73	1.61, 5.41
Describing	27.0	5.4	29.2	6.2	.020	0.38	0.37, 3.96
Acting with awareness	24.4	4.1	26.4	4.6	.078	0.46	0.24, 4.24
Non-judging of inner experiences	27.4	5.6	28.5	4.9	.310	0.21	1.03, 3.12
Non-reactivity to inner experiences	21.0	3.8	24.3	3.0	<.001	0.97	1.65, 5.16
Self-Compassion (SCS)	4.2	1.0	4.7	0.8	.014	0.48	0.09, 0.75
Self-kindness	4.0	1.2	4.6	1.2	.014	0.50	0.13, 1.09
Self-judgment	4.1	1.3	3.6	1.2	.071	0.40	−1.06, 0.05
Common humanity	4.1	1.2	4.5	1.0	.123	0.36	−0.11, 0.89
Isolation	3.5	1.5	3.2	1.3	.133	0.21	−0.74, 0.10
Mindfulness	4.6	1.1	4.8	0.9	.318	0.20	−0.27, 0.79
Over-identification	3.7	1.2	3.2	1.1	.010	0.45	−0.77, 0.11

*BDI* Beck Depression Inventory, *MSQoL-54* multiple sclerosis quality of life–54, *CIS-F* Checklist Individual Strength–Fatigue, *FFMQ* Five Facet Mindfulness Questionnaire, *SCS* Self-Compassion Scale, *CI* confidence interval

### Cognitive Functioning

The majority of patients were not cognitively impaired as compared to the standardized population mean of all tests. Some patients scored below population average on particular tests, and one patient scored below population average (*z* score < −2.00) on all pre-intervention measures, except on the PASAT. Results from paired-samples *t* tests with or without this patient however did not reveal any differences. All test-descriptives and results for paired *t-* tests are presented in Table 3.

**Table 3:** Effects of MBSR on subjective and objective cognitive measures from pre- to post-intervention in 16 MS patients

	Pre-intervention		Post-intervention		*p*	Cohen’s *d*	95% CI
	*M*	SD	*M*	SD			
Working memory							
RAVLT (average words 5 trials)	9.3	2.2	9.7	2.1	-.187	0.21	−1.14, .24
RAVLT delayed recall	9.8	3.8	10.1	2.5	.491	0.12	−1.51, .76
Visuospatial processing							
LLT (average displacement errors 5 trials)	4.9	4.2	3.3	2.7	.042*	0.45	.65, 3.11
LLT delayed recall	1.7	3.8	0.3	2.0	.162	0.49	−62, 3.37
LLT learning index	0.5	.27	0.6	0.3	.071^†^	0.42	.01, −1.94
Processing speed and working memory							
PASAT	46.8	9.5	49.8	10.0	.138	0.30	−6.94, 1.06
Working memory and executive functioning							
Digit span	7.2	1.1	7.3	1.6	.790	0.07	−.83, .64
Letter-number sequencing	10.1	2.7	9.7	3.0	.491	0.13	−.76, 1.51
Language and semantic memory							
Letter fluency (average 3 trials)	11.4	3.4	12.5	3.2	.121	0.34	−2.58, .33
Subjective memory MMQ^a^							
Contentment	45.7	12.2	49.1	13.4	.152	0.26	−8.05, 1.39
Daily forgetfulness	54.7	10.8	56.1	12.0	.422	0.13	−5.27, 2.34
Strategies	24.5	10.3	26.9	9.2	.241	0.25	−6.60, 1.80

Parallel versions at post-intervention were used for RAVLT, LLT, and letter fluency. Higher scores indicate improved performance except for the LLT, which is reversely scored. *RAVLT* Rey Auditory Verbal Learning Test, *LLT* location learning task, *PASAT* Paced Auditory Serial Addition Test, *MMQ* Multifactorial Meta Memory Questionnaire, *CI* confidence intervals

*p < .05, ^†^ *p* < .05, one-tailed, ^a^ *n* = 15

#### Subjective Self-Reported Memory

Patients (*n* = 15) reported an average satisfaction with their memory (*M* = 45.7), an average amount of daily forgetfulness (*M* = 54.7), and reported to use an averaged number of strategies (*M* = 24.5) pre-intervention. There were no significant differences in these reports between pre- and post-intervention. At individual scoring level, 53.3% of the patients (*n =* 8) were more content and satisfied with their own memory after intervention, 60% (*n =* 9) reported less daily forgetfulness, and 73.3% (*n =* 11) reported to use more strategies.

#### New Learning and Verbal Memory

Memory performance at RAVLT did not significantly increase: patients did not remember more words on average at post-test. At individual level, two patients scored below population average (*z* score < −2.00) at the immediate learning trials at pre-test, they improved at post-intervention testing.

#### Visuospatial Memory

Patients made significant less displacement errors at the immediate recall on the LLT at post-test then at pre-test (*t*(15) = 2.22, *p* = .042, *d* = .45). No such improvements were found for delayed recall scores from pre- to post-intervention. Patients showed no relative improvement between five consecutive learning trials, as indicated with the learning index. At individual level, three patients scored below population average (*z* score < −2.00) at the immediate recall and all improved at post-test. Three patients revealed very low scores on the learning index (*z* score < −2.00), which indicates overall improvement on each consecutive trial. They all showed improvement in learning at post-test. One patient decreased overall learning at post-test (*z* score < −2.00).

#### Information Processing Speed

Patients showed no significant improvement on the number of correct repeated calculations at post-test on the PASAT. One patient repeated few calculations (*z* score < −2.00), two patients decreased in performance at post-test, and repeated less calculations than at pretest (*z* score < −2.00).

#### Working Memory and Attention

The total amount of repeated sequences forwards and backwards did not show any significant differences on the digit span test, nor when looking separately at the data for either forward or backwards repeated digits. Two patients repeated a very low amount of total digits (*z* score < −2.00) at pre-test, one improved its performance, and the other patients’ performance remained stable. Another patient decreased its performance at post-test (*z* score < −2.00).

#### Executive Function

Patients did not show improvement at post-test in the total amount of repeated digits on the letter-number sequencing task. Two patients repeated a lower number of digits at post-test (*z* score < −2.00) then at pretest.

#### Language and Semantic Memory

No increased word production was found post-intervention for the letter fluency test. Two patients had low word production at pretest (*z* score < −2.00), both improved at post-test.

## Discussion

The aim of this pilot study was to examine the effectiveness of MBSR on psychological and cognitive functioning in patients with MS, using self-report questionnaires and objective cognitive tests. The results suggest that after participation in MBSR, patients experienced less depressive symptoms, improved quality of life, both in the physical as well as the mental domain, and patients were less fatigued. Patients also improved in their mindfulness skills. They were better able to observe and describe their experiences, and were less likely to automatically react to inner experiences. Moreover, patients improved in self-compassion, such that they were kinder towards themselves and were less likely to identify with negative thoughts, emotions, or sensations. The largest effects were found for the improved quality of life measures. These findings replicate two former randomized controlled trials that showed beneficial effects of MBI on psychological distress in MS patients (Bogosian et al. 2015; Grossman et al. 2010). In contrast to previous studies, some of the patients in the current study were cognitively impaired. Due to this, the present study was able to demonstrate that the presence of cognitive impairments does not determine the positive outcome on reduction in psychological distress after MBSR.

There were hardly any improvements, however, in cognitive functioning nor in subjective memory reports. Patients did improve in visuospatial memory processing, but not in verbal memory, processing speed, and working memory, nor in executive functions. For those subjects that were cognitively impaired at pre-test level, there were some individual benefits at post-test. Also on individual level, there was a trend that patients reported more memory satisfaction, less daily forgetfulness, and more memory-strategy use, after the intervention. These findings should be seen in the light of research showing that once cognitive dysfunction occur in MS patients, it is unlikely to remit to any significant extent. It might remain stable but is more often progressive at variable rates of deterioration (Amato et al. 2006). Thus, although improvements might be small, they might be of relevance to patients. These findings seem in line with a recent systematic review, showing the beneficial impact of MBIs on cognitive functioning (Chiesa et al. 2011). However, improvements on cognitive tasks, similar to the ones used in the present study, were mostly found in healthy participants and not in patients with chronic neurological diseases. Interestingly, Larouche et al. (2015) speculated that MBIs could have a potential effect on age-related cognitive decline and could delay onset of Alzheimer’s disease, as it alleviates modifiable risk factors such as stress, depression, and inflammation. In line with this speculation, we could hypothesize that for the MS population this means that the beneficial effects of MBSR on psychological distress could indirectly have a beneficial effect on cognitive functioning (see also Chiesa et al. 2011).

### Limitations and Strengths

Several limitations of this pilot study should be noted. First, as this is an exploratory pilot study, no control group was used. Therefore, findings cannot directly be attributed to the MBSR. As this study demonstrates positive effects of MBSR on psychological well-being, and potential effects on cognitive functioning, future studies should include a control group. Second, the relatively high dropout rate resulted in a small sample size, limiting the power of the analyses. This means that the chance of a type II error occurring is heightened, and the estimates of effect size are less reliable. The relatively high dropout rate could be explained by the study being embedded in clinical practice. The research support was limited, and little time could be spent on retaining patients in the trial. Future research should include a larger sample of patients and compare MBSR with a control group. Third, no detailed inclusion criteria were applied, resulting in a rather heterogeneous patient group. MS is rather complicated in its manifestation of symptoms and illness progression, which may complicate interpretation and generalization of this study. Unfortunately, due to the small sample size, we were unable to examine whether type of MS or illness duration predicted outcome. Future trials with larger sample sizes should examine these prognostic factors to help answer the question which group of patients may particularly benefit from MBSR. Moreover, researchers should consider using these factors to stratify randomization in future trials. In addition, patients were not included based on cognitive functioning. To find improvements in cognitive functioning, future studies could consider including only patients with cognitive impairment. Currently, we are preparing an RCT that will examine the effects of an MBI in MS patients with cognitive impairments. Fourth, unfortunately we did not extensively examine how feasible MBSR participation is in patients with MS. Future studies should not only examine those who eventually participate in the study but also those who refuse and list the number of eligible and willing patients. Moreover, the number and reasons for refusing participation or dropping out the intervention should be registered. This data could inform practitioners about how feasible MBSR participation is in MS patients, and how it could be implemented in clinical practice. Last, psychological outcomes and subjective memory complaints were only based on self-reports, which may lead to biased results. Future studies could strengthen outcomes that are based on self-reports by questioning patients’ relatives.

This study also had a number of strengths. Only two identified studies have examined the effects of MBSR on psychological well-being and quality of life in MS patients. This study included cognitive functioning as an outcome, suggesting that MBSR might improve visuospatial memory processing in MS patients. Moreover, this study that included measures of mindfulness skills and self-compassion, which are seen as two of the key ingredients of MBIs (Gu et al. 2015). Although the validity of these measures is still under debate (Grossman and van Dam 2011; Muris and Petrocchi 2016), the scales have high internal consistency and have been adopted successfully in studies on the effects of MBIs (Baer 2011; Neff 2016). In addition, the reported effect sizes in the present study can be used for power calculation essential for future studies.

To conclude, this pilot study demonstrated improved psychological well-being in MS patients after MBSR and suggests it can improve visual-spatial processing. The positive effects on psychological well-being suggest that MBSR might be valuable for patients with neurological diseases, even for those that are cognitively impaired. Since cognitive deficits—but also psychological distress—could be devastating for patients with MS, especially this group of patients should be targeted for effective care. Future randomized controlled trials should be conducted to confirm the possible effectiveness of MBSR—and its long-term effects—on psychological and cognitive functioning in MS patients.
